# Early economic evaluation of chelation therapy in kidney transplant recipients with high-normal lead

**DOI:** 10.1371/journal.pone.0319022

**Published:** 2025-02-27

**Authors:** Jiasi Hao, Behrooz Z. Alizadeh, Maarten J. Postma, Daan J. Touw, Stephan J. L. Bakker, Lisa A. de Jong

**Affiliations:** 1 Department of Epidemiology, University Medical Center Groningen, University of Groningen, Groningen, The Netherlands; 2 Department of Health Sciences, University Medical Center Groningen, University of Groningen, Groningen, The Netherlands; 3 Department of Economics, Econometrics and Finance, Faculty of Economics and Business, University of Groningen, Groningen, The Netherlands; 4 Center of Excellence for Pharmaceutical Care Innovation, Universitas Padjadjaran, Bandung, Indonesia; 5 Department of Clinical Pharmacy and Pharmacology, University Medical Center Groningen, University of Groningen, Groningen, The Netherlands; 6 Department of Nephrology, University Medical Center Groningen, University of Groningen, Groningen, The Netherlands; Phramongkutklao College of Medicine, THAILAND

## Abstract

**Background:**

Kidney transplant recipients (KTR) with high-normal lead have a higher risk of graft failure (GF). Clinically, chelation therapy using meso-2,3-dimercaptosuccinic acid (DMSA) removes lead. Despite the proposal that chelation therapy can prevent GF through lead removal, evidence is lacking. To guide research efforts, we conducted an early economic evaluation, aiming to explore the economic feasibility of screening for and implementing chelation therapy with oral DMSA for high-normal plasma lead concentrations in KTR (i.e., the intervention) compared to standard of care.

**Methods:**

A Markov model simulated the life course of 10,000 KTR in the Netherlands from a societal perspective. Transition probabilities were estimated using the data from TransplantLines Food and Nutrition Biobank and Cohort study. Costs and utilities were sourced from publications and public data. Model robustness was investigated through deterministic and probabilistic sensitivity analyses. Various administration strategies were tested. Five-year costs were calculated from a healthcare payer’s perspective. Value of information was assessed.

**Results:**

The intervention was cost-saving and improved health, leading to a dominant incremental cost-effectiveness ratio. The result was most sensitive to transition probabilities (led by GF, followed by death with functioning graft and after graft failure). The probability of the intervention being cost-effective was 60%. Chelation strategies did not affect the result. The intervention applied to the Dutch KTR population could save €27 million in the initial five years. Further research is desirable if the cost of obtaining perfect information on GF survival is approximately below €4,000/KTR (all uncertainties under €5,000/KTR).

**Conclusion:**

The cost-effectiveness of the intervention is robust in KTR, except when considering the uncertainties around (graft) survival probabilities. Applying chelation therapy in the new setting we studied holds significant potential. However, trials that systematically assess the efficacy, administration strategies, and impacts on survival are crucial in updating the current evaluation and informing policies.

## Introduction

Lead is a toxic heavy metal predominantly found in environmental exposures, including ambient air, soil, dust, and various consumer products [1–3]. In the Netherlands, primary lead exposure originates from food and tap water supplied through ageing lead pipes [3–6], with additional contributions from legacy industrial lead and imported goods such as cosmetics [3]. Different from acute poisoning, where lead concentration surges over a dangerously high threshold, chronic exposure—from lifestyle factors, occupational settings and living conditions—poses a widespread public health concern [3,7]. Extensive evidence has indicated that even low-level chronic lead exposures can cause lead accumulation first in bones and then in kidneys [7,8], further contributing to nephrotoxicity and kidney damage [7]. As one of the risk factors for chronic kidney disease (CKD) among obesity, hypertension and diabetes mellitus [7,9], lead exposure and its harms have been overlooked. A recent observational study from Sotomayor et al. (2022), in which a cohort of kidney transplant recipients (KTR) was trichotomized based on plasma lead concentrations, demonstrated that higher plasma lead concentrations at a non-toxic level (high-normal plasma concentrations, i.e., high group), are independently and consistently associated with an increased risk of long-term graft failure [10,11]. Recognizing the detrimental impact of lead on kidney function, proposals as early as the 2000s recommended screening CKD patients for acute toxic and chronic high-normal heavy metal levels, including lead [12,13].

CKD is a gradual decline in kidney function that persists for over three months. It has five stages based on the estimated glomerular filtration rate (eGFR). According to the Kidney Disease Improving Global Outcomes (KDIGO) foundation guidelines, end-stage kidney disease (ESKD) occurs when the kidneys can no longer effectively filter waste products from the blood for survival (i.e., eGFR < 15 ml/min/1.73 m^2^) [14]. Treatment for ESKD is limited to kidney replacement therapy—dialysis and/or kidney transplantation. Dialysis, a medical procedure that mimics normal kidney functions to filter waste products, can be performed either externally through machines (haemodialysis [HD]) or internally through the body (peritoneal dialysis [PD]). Modalities vary the costs of €95–€129 thousand annually per patient in the Netherlands [15]. Kidney transplantation, a surgical transfer of a healthy kidney from a donor into a patient, incurs initial costs of €56–€152 thousand; when successful, its subsequent annual upkeep expenses are substantially lowered [15]. Regardless, sustaining kidney function is essential at every stage. In addition to improving quality of life and patient experience, this is critical because kidney replacement therapy is expensive, and transplant failure would require dialysis or re-transplantation, placing further strain on resource-scarce healthcare system [16].

Acute lead poisoning is treated by chelation therapy [17], which uses chelating agents to bind metals and facilitate their removal through urine or feces. However, the efficacy of chelation therapy in reducing high-normal lead and its potential to prolong graft function through lead removal in CKD are mostly unknown. Earlier trials have shown that repeated low-dose chelation therapy with chelator calcium disodium ethylenediaminetetraacetic acid (CaEDTA) effectively slows kidney function decline in CKD patients with low-level lead exposure measured by blood [18–21], which is effective over four years [22]. Despite positive results, these efforts have not been followed up or updated with technologies. For instance, compared to blood, lead measurement from plasma has a lower detection limit, higher sensitivity and better precision [23]. A newer chelator, meso-2,3-dimercaptosuccinic acid (DMSA), is currently preferred and commonly used due to reduced disruption of essential minerals, including calcium, copper, and zinc, and fewer side effects [24–27]. Its potential efficacies and possible oral capsule administration, relative to kidney replacement therapy, could theoretically delay the progression to graft failure and thus intuitively reduce healthcare costs in CKD. This may be particularly true for KTR. As the transplanted kidneys of KTR, compared to functioning native kidneys, face a heavier functional burden and demonstrate a greater susceptibility due to ischaemia-reperfusion injury during the transplantation procedure and intrinsically nephrotoxic immunosuppressants used post-transplantation, any means of reducing additional nephrotoxicity from these vulnerable transplanted kidneys could be essential to preserve graft function.

In this study, we aimed to evaluate the economic feasibility and provide considerations for using oral DMSA to prevent graft failure in KTR with high-normal plasma lead concentrations compared to the standard of care. Given the early stage of the intervention (i.e., chelation therapy using DMSA) in a new setting (i.e., KTR) and a new treatment goal (i.e., maintaining graft functioning), we conducted an early economic evaluation aligned with guidance from The Early Health Technology Assessment (HTA) report drafted by the Dutch HTA consortium [28]. Using patient-level data from the University Medical Center Groningen (UMCG), the Netherlands, we performed analyses on cost-effectiveness, budget impact and value of information. Particularly pivotal, the report also emphasizes the exploratory nature of early HTA [28]. While our findings are likely not definitive or policy-prescriptive, they provide a solid foundation and invaluable insights to guide and inform future research.

## Materials and methods

An early economic evaluation was performed, which comprised cost-effectiveness, budget impact analyses and value of information. Incremental costs (ΔC) and incremental effectiveness (ΔE, i.e., quality-adjusted life years [QALYs] calculated from utilities) for the intervention compared with the standard of care were calculated. The primary outcome is the incremental cost-effectiveness ratio (ICER = ΔC/ΔE). The analyses were conducted in accordance with the Dutch guideline for economic evaluation in healthcare and reported in line with the Consolidated Health Economic Evaluation Reporting Standards (CHEERS) 2022 checklist (S1 Table). Analyses were conducted in RStudio, version 2022.12.0 + 353 [29].

### Patient cohort

We used the data on adult KTR at the UMCG who participated in the TransplantLines Food and Nutrition Biobank and Cohort study (NCT03272841) [30], which is the same cohort in which it was found that elevated plasma lead concentrations were associated with an increased risk of graft failure [11]. The participants were recruited between November 21, 2008 and May 24, 2011. All participants provided written informed consent on enrollment. The study protocol received approval from the Medical Ethical Committee (Medisch Ethische Toetsingsingscommissie [METc]) of the UMCG (METc 2008/186) and was conducted in compliance with the Declaration of Helsinki.

The baseline characteristics of the hypothetical cohort and lead-concentration-based (graft) survival curves were based on an actual cohort size of 670 subjects with available plasma lead measurements. The plasma lead concentration was measured at admission for kidney transplantation and 3-, 6-, 12- and 24-months post-transplantation. Due to the lead stability that was found over this two-year follow-up period [11], the values at the admission were used, with a median of 0.31 µg/L (interquartile range [IQR]: 0.22–0.45 µg/L, distribution shown in S1 Fig). The trichotomized low, medium, and high groups had a mean plasma lead concentration of 0.19, 0.31, and 0.65 µg/L (S2 Table).

### Model description

**Fig 1:**
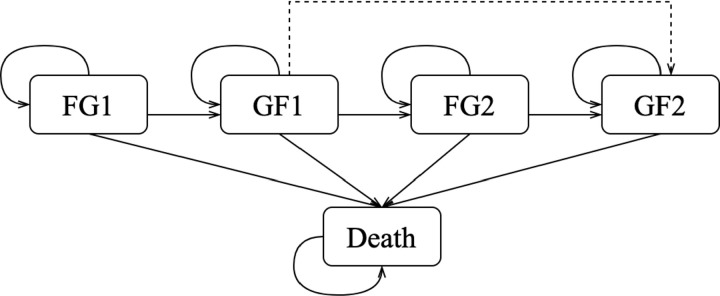
Markov model for progression of disease. The dashed arrow indicates primary non-function. From each state, it is possible to transition to the death state. FG1, functioning graft 1; FG2, functioning graft 2; GF1, graft failure 1; GF2, graft failure 2.

A Markov model was developed to simulate the life course of KTR. A lifetime horizon (40 years from the first kidney transplant) and a one-year cycle length were used. The model, structured similarly to the one from Coerts et al. (2021) [31], consists of three health states which are functioning graft (FG), graft failure (GF), and death (Fig 1). A hypothetical cohort of 10,000 KRT entered the model at the FG1 stage. Since these patients still have options for re-transplantation if the transplanted kidney fails (from FG1 to GF1), a second transplantation was incorporated into the model (from GF1 to FG2). We assumed a maximum of two transplantations as a third transplantation is rare [32]. Every transplantation has a chance of primary non-function, which refers to an immediate and persistent functioning failure of a transplanted kidney (from GF1 to GF2) [33]. Each state has a chance of death, which can be categorized into death with functioning graft (DWFG) and death with graft failure (DwGF). Trapezoidal half-cycle correction was applied to account for events occurring at any point within a cycle [34].

### Standard of care and chelation therapy

The standard of care post-transplantation is regular checkups, including blood tests, every three months at a scheduled outpatient visit. There is no screening for heavy metals. The intended intervention—chelation therapy—includes 1) initial screening of all incident KRT through an additional order of plasma lead measurement from the blood sample taken at the first post-transplantation regular checkup, 2) providing oral DMSA to the eligible KTR, and 3) among these eligible KTR, conducting follow-up screening in case of lead re-exposure every five years, using the blood sample taken via the standard of care, and administering oral DMSA if re-exposed.

The administration of DMSA can be oral or intravenous. Considering the minor elevation in plasma lead concentration among KTR, oral DMSA was more suitable for our study and is more convenient to be administered at home. Oral DMSA consists of one or multiple courses of treatment, with each course comprising five days of capsule intake and a one-month waiting period for lead redistribution in the body, followed by a blood test to assess the results and determine if an additional course is needed.

Blood tests were used for screening and monitoring in the process of chelation therapy; thus, their timing is important. The initial screening was conducted upon inclusion in the Markov process, and the follow-up screenings were conducted after the re-exposure period. The the lead removal effect was monitored at the beginning of the second year of chelation therapy and chelation therapy may follow depending on the results.

**Fig 2:**
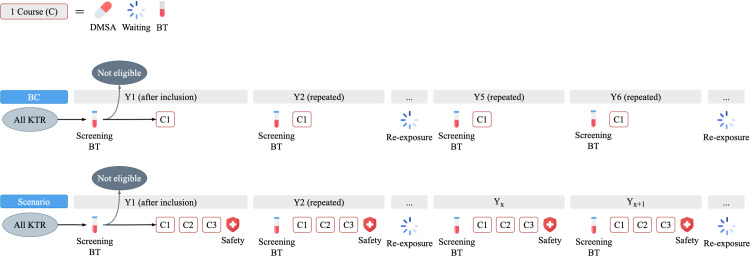
The timeline and procedure of screening and implementing chelation therapy in base case. C, Course; C1, Course 1 (i.e., one course); DMSA, meso-2,3-dimercaptosuccinic acid; BT, blood test; KTR, kidney transplant recipient; Y, year.

The timeline and procedure of the intervention in the base case (BC) are visualized in Fig 2. The assumptions were as follow: a1) only the KTR in the high group were eligible for chelation therapy to achieve interventional benefits while avoiding potential side effects (i.e., intervention threshold 0.38 µg/L); a2) chelation therapy would help the eligible KTR to have a lowered lead burden from high to medium group (i.e., stopping threshold 0.38 µg/L); a3) no adverse event; a4) the eligible KTR would not require chelation therapy for at least five years after the first course of repeated chelation therapy; and afterwards, a5) in the follow-up screening every five years, 10% of the initial eligible KTR, would have a plasma lead concentration exceeding the intervention threshold, and thus, require another chelation therapy. Additionally, a6) lead stability was assumed, meaning that all KTR would remain in the same group except for the KTR eligible for chelation therapy who would transition from the high (medium) to medium (high) group due to the intervention (re-exposure). S3 Table elaborates on the rationales behind these assumptions and their potential impacts on results. Overall, the assumptions were deliberately conservative to ensure study validity and alignment with the study aims.

### Transition probabilities

The transition probabilities were derived from the UMCG dataset and literature (Table 1). To estimate and extrapolate the transition probabilities for GF, DWFG and DwGF, parametric survival modeling was applied in compliance with the National Institute for Health and Clinical Excellence (NICE) guideline [35,36]. Models were fitted using the UMCG cohort with distributions including Exponential, Weibull, Gompertz, Gamma, Log-normal, Log-logistic, and Generalized Gamma. Model selection was based on visual inspection, Akaike information criterion (AIC), Bayesian information criterion (BIC), and expert opinions (co-author L.A.J. and S.J.L.B.) to ensure both methodological rigor and clinical applicability. Parameters from the best-fit models (i.e., GF: Log-normal, DWFG: Gamma, and DwGF: Gamma) were then used to extrapolate the survival curves to the model’s time horizon (S4 Table and S2 Fig), from which transition probabilities were subsequently calculated ($transitionprobability_{i}=1-survivalprobability_{i}/survivalprobability_{i-1}$, where *i* denotes time in years). For the GF endpoint (transitions from FG1 to GF1, and from FG2 to GF2), the parametric survival analysis was performed separately for each of the three lead groups at low, medium and high levels, adjusting for age, sex, transplant vintage, estimated glomerular filtration rate (eGFR) and 24-hour urinary protein excretion, to approximate the efficacy of oral DMSA in prolonging graft functioning through reduced lead. For DWFG (from FG1 and FG2 to Death) and DwGF (from GF1 and GF2 to Death) endpoints, the analyses were conducted in all KTR without lead trichotomization.

**Table 1:** Parameters.

Variable	States	Value	Range	Source
*A. Transition probabilities*				
GF	FG1 to GF, FG2 to GF2			
Low plasma lead		S4 Table and S2A Fig		UMCG dataset [30]
Medium plasma lead		S4 Table and S2A Fig		UMCG dataset [30]
High plasma lead		S4 Table and S2A Fig		UMCG dataset [30]
DWFG	FG1 to Death, FG2 to Death	S4 Table and S2B Fig		UMCG dataset [30]
DwGF	GF1 to Death, GF2 to Death	S4 Table and S2C Fig		UMCG dataset [30]
Re-transplantation	GF1 to FG2	S2D Fig		Groen et al. [37]
Age of starting decline		65	NA	Assumption
Age of declining to zero		80	NA	Assumption
Primary non-function	GF1 to GF2	0.007	0.004–0.010	UMCG dataset [30]
*B. Costs and productivity losses*				
Costs for chelation therapy				
Drawing blood	NA	€15.00	€7.65–€22.35	Price List for Mutual Services, multiple UMCs [38–40]
Lab result	NA			
Lead		€50.63	€25.82–€75.43	Price List for Mutual Services, UMCG [41]
Iron^a^		€26.39	€13.46–€39.32	Price List for Mutual Services, UMCG [41]
AST, ALT^a^		€5.38	€2.74–€8.02	Price List for Mutual Services, UMCG [41]
DMSA capsules^b^	NA	€570.95	€291.18–€850.72	Department of Purchase of Medicines, UMCG [42]
Oral iron supplements^a^	NA	€46.48	€23.70–€69.26	Dutch Pharmacotherapeutic Compass [43]
Costs per health state				
Graft failure initial costs	FG1 to GF1, FG2 to GF2	€2,818.80	€1,437.59–€4,200.01	Groen et al. [37]
Dialysis upkeep	GF1, GF2			
CHD		€113,142.50	€61,662.98–€164,621.94	Mohnen et al. [15]
HHD		€106,344.10	€76,059.79–€136,628.39	Mohnen et al. [15]
APD		€109,863.60	€64,633.50–€155,093.72	Mohnen et al. [15]
CAPD		€94,756.94	€29,540.82–€159,973.05	Mohnen et al. [15]
% CHD		80.0%	NA	Renine Registry Annual Report 2021 [44]
% HHD		4.0%	NA	Renine Registry Annual Report 2021 [44]
% APD		8.0%	NA	Mohnen et al. & Renine Registry Annual Report 2021 [15,44]
% CAPD		8.0%	NA	Mohnen et al. & Renine Registry Annual Report 2021 [15,44]
Transplantation	GF1 to FG2	€103,993.68	€8,986.50–€199,000.85	Mohnen et al. [15]
Transplantation upkeep	FG1, FG2	€14,376.98	€7,332.26–€21,421.70	Groen et al. [37]
Death	Death	€1,392.03	€709.93–€2,074.12	Groen et al. [37]
Productivity losses				
% KTR working after transplantation	GF1 to FG2			
Age 45–54		57.0%	29.1%–84.9%	Jansen et al. [45]
Age 55–64		41.0%	20.9%–61.1%	Jansen et al. [45]
Age 65 +		6.1%	3.1%–9.1%	Assumption (Same decline as in general population)
% KTR working under dialysis	FG1 to GF1, FG2 to GF2			
Age 45–54		32.0%	16.3%–47.7%	Jansen et al. [45]
Age 55–64		19.0%	9.7%–28.3%	Jansen et al. [45]
Age 65 +		2.8%	1.4%–4.2%	Assumption (Same decline as in general population)
Days in hospital	GF1 to FG2			
Living donor		13.30	6.78–19.82	UMCG Kidney Transplant Annual Report [46]
Deceased donor		7.80	3.98–11.62	UMCG Kidney Transplant Annual Report [46]
Friction period, day	NA	85.00	43.35–126.65	Dutch National Healthcare Institute [47]
Working hours per day	NA	8.00	4.08–11.92	Dutch National Healthcare Institute [47]
Hourly salary	NA			
Male		€28.45	€23.47–€68.57	Statistics Netherlands [48]
Female		€24.70	€19.57–€57.17	Statistics Netherlands [48]
Discounting				
Costs	NA	4%	0.0%–8.0%	Dutch National Healthcare Institute [47]
*C. Utilities per health state*				
Functioning graft	FG1, FG2	0.81	0.72–0.90	Liem et al. [49]
Graft failure	GF1, GF2			
HD		0.56	0.49–0.62	Liem et al. [49]
PD		0.58	0.50–0.67	Liem et al. [49]
Death	Death	0.00	0.00	NA
Discounting				
Effect	NA	1.5%	0.0%–3.0%	Dutch National Healthcare Institute [47]

^a^Cost items only used in scenario analysis.

^b^Cost item calculated based on purchasing price, average weight in the cohort, tax, and assumed dose.

GF, graft failure; DWFG, death with functioning graft; DwGF, death with graft failure; FG1, functioning graft 1; FG2, functioning graft 2; GF1, graft failure 1; GF2, graft failure 2; CHD, center haemodialysis; HHD, home haemodialysis; APD, automated peritoneal dialysis; CAPD, continuous ambulatory peritoneal dialysis; KTR, kidney transplant recipients; FG, functioning graft; GF, graft failure; UMCs, University Medical Centers; UMCG, University Medical Center Groningen; Renine, Dutch Renal Registry; HD, haemodialysis; PD, peritoneal dialysis.

The probability of re-transplantation (from GF1 to FG2) was based on literature [37], which was assumed to linearly decline from 0.15 after age 65 and reach zero at the age in the last cycle. The probability of non-primary (from GF1 to GF2) was assumed to be 0.7%, accounting for the patients with a third or fourth kidney in the UMCG cohort.

### Costs

The societal perspective requires both direct medical costs and indirect costs (Table 1). All costs were adjusted for inflation using the consumer price index (CPI) from Statistics Netherlands (Centraal Bureau voor de Statistiek [CBS]) and presented in EUR 2022 [50]. Whenever possible, cost data were obtained from the Dutch costing manual for economic evaluations in healthcare [47].

Costs associated with chelation therapy were based on published price lists from the Dutch university medical centers and expert advice (co-author D.J.T.). Blood sampling costs, not available on the UMCG website and slightly varying across centers, were averaged. Given the lack of evidence on chelation therapy in KTR with high-normal lead concentrations, a low dose of 10 mg/kg/day was assumed [51,52]. According to the National Institute for Public Health and the Environment (Rijksinstituut voor Volksgezondheid en Milieu [RIVM]) in the Netherlands, DMSA is an unlicensed drug with no domestic stock or suppliers. Hence, the capsules must be imported. The purchasing price of DMSA Succicaptal^®^ 200 mg (15 capsules) for hospital pharmacists was €400.47, excluding 6% value-added tax (VAT) [42]. Blood tests for chelation therapy can be combined with regular outpatient visits every three months, meaning no additional costs concerning travel or outpatient services. Therefore, all procedures involved in chelation therapy can be considered an add-on to the standard of care.

Costs per health state reflect those directly incurred by a patient being in a health state. For example, in the graft failure state (GF1, GF2), initial graft failure and ongoing dialysis costs were included. Dialysis costs depended on the chosen modality: Conventional center HD (CHD) requires hospital visits and trained professionals, while home HD (HHD) involves patients being trained to use a portable dialysis machine at home. Automated PD (APD) allows for automatic filling and draining of dialysate, while continuous ambulatory PD (CAPD) requires manual dialysate change. Costs for transplantation and post-transplantation care were applied when a patient transitioned to or remained in the functioning graft state (FG1, FG2). One-off costs for death occurred when a patient transitioned to the death state. Following the Dutch guideline for economic evaluation in healthcare, productivity losses were estimated using the friction cost method.

Annual costs were summed for each Markov trace and discounted at a 4% rate.

### Utilities

Utility values for each health state were based on European Quality of Life, Five-dimension, Three-level questionnaire (EQ-5D-3L) values reported in a systematic review and meta-analysis (Table 1). In the graft failure states (GF1, GF2), the utility value was calculated in proportion of patients on HD (84%) and PD (16%). The utility for death was assumed to be zero. For each Markov trace, the annual QALYs were summed up and discounted at a rate of 1.5%.

### Sensitivity analyses

To assess the robustness of the model and results, univariate deterministic sensitivity analysis (DSA), multivariate probabilistic sensitivity analysis (PSA), and scenario analyses were performed. In DSA, one parameter was varied at a time to the lowest and highest value of the 95% confidence interval (CI), with unavailable CI assumed as a standard error of 25% of the parameter’s value. Additionally, considering the high uncertainty in the price of DMSA capsules, variations of 50% reduction and 200% increase were tested. The results of the DSA analysis were plotted in a tornado diagram.

In PSA, all parameters were varied simultaneously by random draws from their corresponding distributions: beta distributions for the utilities and the percentages of the working population; a gamma distribution for all the costs; and a normal distribution for the number of courses, time spent in hospital, the probabilities of re-transplantation and non-primary function. The uncertainties in the transition probabilities for GF, DWFG, and DwGF were incorporated by random draws from normal distributions defined by parameter estimates and 95% CIs of the chosen best-fit models (S4 Table and S2 Fig). Monte Carlo simulations were performed with 10,000 iterations and plotted in a cost-effectiveness plane with a willingness-to-pay (WTP) threshold of €50,000/QALY, as recommended by the Dutch National Healthcare Institute considering the burden of disease (proportional shortfall: 0.63) [53,54]. To assess the probability of being cost-effective at different WTP thresholds, the simulation results were plotted in a cost-effectiveness acceptability curve (CEAC).

Scenario analyses were conducted to overcome the uncertainties surrounding chelation therapy. Key assumptions, such as transplant vintage, time horizon, discounting, and inflation rates, were examined. The exploratory nature of the study prompted scenario analyses to investigate the intervention strategies, including the number of courses, effect waning and frequency of conducting follow-up screenings, and subsequent intervention (Table 2). Adverse events, although from the previous evidence unlikely to occur [52,55], mainly include e1) impact on other metals, e2) transaminase activities, and e3) skin reactions [51]. These adverse events are either inconclusive (e1) or naturally dissolved after treatment discontinuation (e2-3), with no significant impacts on patients, especially given the low DMSA dosage in our study. However, considering the vulnerability of KTR, two safety measures, checking for iron deficiency (e1) and liver dysfunction (e2), were added to the scenarios so that any adverse effects could be detected and acted on in time, ensuring patient safety.

**Table 2:** Scenarios.

Scenario	Other assumptions	Screening		Chelation therapy (1st year)			Chelation therapy (2nd year)		Safety measures			Re-exposure		
		BT^a^	Threshold	C1	C2	C3	BT	Repeated	Iron deficiency	Oral iron supplement	Liver function	Time, year	High again	Repeated
BC	Time horizon, lifetime Discounting costs, 4.0% Discounting effects, 1.5%	Yes	High	100%	0%	0%	Yes	60%	No	No	No	5	10%	Every 5 years
1	**Time horizon, 10 years**	Yes	High	100%	0%	0%	Yes	160%	No	No	No	5	10%	Every 5 years
2	**Time horizon, 20 years**	Yes	High	100%	0%	0%	Yes	260%	No	No	No	5	10%	Every 5 years
3	**Discounting costs, 0%**	Yes	High	100%	0%	0%	Yes	360%	No	No	No	5	10%	Every 5 years
4	**Discounting effects, 0%**	Yes	High	100%	0%	0%	Yes	460%	No	No	No	5	10%	Every 5 years
5	Same as BC	Yes	High	100%	**30%**	0%	Yes	60%	No	No	No	5	10%	Every 5 years
6	Same as BC	Yes	High	100%	**70%**	0%	Yes	60%	No	No	No	5	10%	Every 5 years
7	Same as BC	Yes	High	100%	**30%**	**50%**	Yes	60%	No	No	No	5	10%	Every 5 years
8	Same as BC	Yes	High	100%	**70%**	**50%**	Yes	60%	No	No	No	5	10%	Every 5 years
9	Same as BC	Yes	High	100%	0%	0%	Yes	**30%**	No	No	No	5	10%	Every 5 years
10	Same as BC	Yes	High	100%	0%	0%	Yes	**80%**	No	No	No	5	10%	Every 5 years
11	Same as BC	Yes	High	100%	0%	0%	Yes	60%	**Yes**	No	No	5	10%	Every 5 years
12	Same as BC	Yes	High	100%	0%	0%	Yes	60%	**Yes**	**Yes, 8 weeks**	No	5	10%	Every 5 years
13	Same as BC	Yes	High	100%	0%	0%	Yes	60%	**Yes**	No	**Yes**	5	10%	Every 5 years
14	Same as BC	Yes	High	100%	0%	0%	Yes	60%	No	No	No	5	**1%**	Every 5 years
15	Same as BC	Yes	High	100%	0%	0%	Yes	60%	No	No	No	5	**30%**	Every 5 years
16	Same as BC	Yes	High	100%	0%	0%	Yes	60%	No	No	No	5	**60%**	Every 5 years
17	Same as BC	Yes	High	100%	0%	0%	Yes	60%	No	No	No	**10**	**1%**	**Every 10 years**
18	Same as BC	Yes	High	100%	0%	0%	Yes	60%	No	No	No	**10**	**10%**	**Every 10 years**
19	Same as BC	Yes	High	100%	0%	0%	Yes	60%	No	No	No	**10**	**30%**	**Every 5 years**
20	Same as BC	Yes	High	100%	0%	0%	Yes	60%	No	No	No	**10**	**60%**	**Every 5 years**
21	Same as BC	Yes	High	100%	0%	0%	Yes	60%	No	No	No	**20**	**1%**	**Every 10 years**
22	Same as BC	Yes	High	100%	0%	0%	Yes	60%	No	No	No	**20**	**10%**	**Every 10 years**
23	Same as BC	Yes	High	100%	0%	0%	Yes	60%	No	No	No	**20**	**30%**	**Every 5 years**
24	Same as BC	Yes	High	100%	0%	0%	Yes	60%	No	No	No	**20**	**60%**	**Every 5 years**
25	Same as BC	Yes	High	100%	0%	0%	Yes	60%	No	No	No	**Lifetime**	**0%**	**NA**

^a^Screening blood test was intended for all incident kidney transplant recipients to screen for the patients who are eligible for chelation therapy. The following blood tests were only intended for patients screened with high plasma lead-level.

BC, base case; BT, blood test; C1-3, course 1-3.

### Budget impact analysis

Budget impact analysis (BIA) assessed the short-term costs or savings of implementing chelation therapy in KTR in the Netherlands. The Dutch KTR population has a prevalence of 12,068 and an incidence of 957, according to the Dutch Renal Registry (Renine) [44]. It was assumed that all KTR would undergo a screening blood test and a third of KTR would be eligible for chelation therapy. A dynamic cohort and five-year time horizon were used with a healthcare payer’s perspective. The budget impact was calculated by the total number of patients multiplied by the discounted cost of the intervention at each cycle per patient.

### Value of information

Value of information (VOI) evaluates whether further research to gather new data is justified by weighing the benefit of reducing uncertainties against its cost. The expected value of perfect information (EVPI), expected value of partial perfect information (EVPPI), and expected value of sample information were calculated in €/KTR across WTP and visualized in plots.

## Results

### Cost-effectiveness analysis

Screening for and implementing chelation therapy with oral DMSA in KTR with a high-normal plasma lead concentration (referred to as “the intervention” below), compared to standard of care, yields cost savings of €57.56 million and QALY gains of 351.88 per 10,000 KTR, thereby a dominant ICER (Table 3).

**Table 3:** Deterministic results of cost-effectiveness analysis in the base case with a cohort of 10,000 KTR.

	Cost category	Costs (million €)	Effectiveness (QALY)	ΔC (million €)	ΔE (QALY)	ICER
Screening and implementing chelation therapy	Health state-related	€1,728.22		−€63.07		Dominant
	Screening and treatment-related	€5.97		€5.97		
	Productivity losses	€40.25		−€0.45		
	Total	€1,774.43	76,878.81	−€57.56	351.88	
Standard of care	Health state-related	€1,791.29				
	Screening and treatment-related	€0.00				
	Productivity losses	€40.70				
	Total	€1,831.99	76,526.93	NA	NA	

QALY, quality adjusted life year; ΔC incremental costs; ΔE incremental effectiveness; NA, not applicable; ICER, incremental cost-effectiveness ratios.

### Deterministic sensitivity analysis

**Fig 3:**
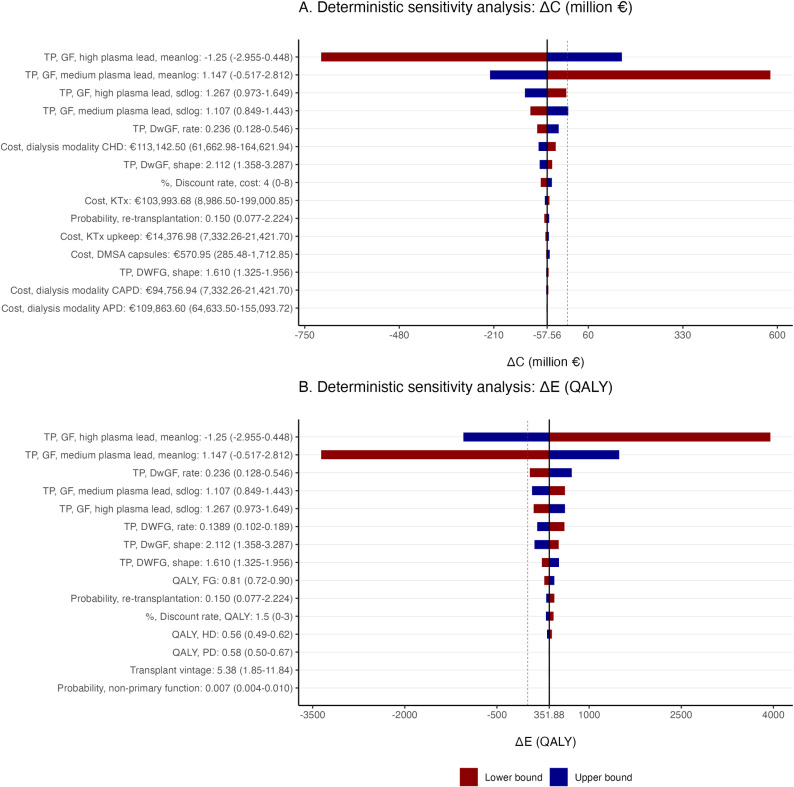
Deterministic sensitivity analysis. The tornado diagram represents the effect of the lower and upper limit of every parameter on (A) incremental costs and (B) incremental effectiveness. The 15 most influential parameters are shown. The red dashed line represents either ΔC=0 or ΔE=0 Meanlog and sdlog are the parameters from lognormal distribution and were estimated through the parametric survival analyses. These parameters are used to calculate the transition probabilities for graft failure in different subgroups of KTR with high, medium and low plasma lead concentrations. ΔC incremental costs; ΔE incremental effectiveness; QALY, quality adjusted life year; TP, transition probability; GF, graft failure; DwGF, death with graft failure; DWFG, death with functioning graft; FG, functioning graft; HD, haemodialysis; PD, peritoneal dialysis; CHD, center haemodialysis; CAPD, continuous ambulatory peritoneal dialysis; KTx, kidney transplantation; DMSA, meso-2,3-dimercaptosuccinic acid.

The 15 most influential parameters are illustrated in Fig 3 (all parameters see S3 and S4 Figs). Among all, the means of logarithm (i.e., meanlog) from lognormal distribution, used to calculate the transition probabilities for GF in the medium and high groups, has the greatest impact on both ΔC and ΔE This is followed by the standard deviations (i.e., sdlog) of logarithm, similarly used for calculating the transition probabilities for GF in medium and high groups, and the rate from gamma distribution used to calculate the transition probability for DwGF. The transition probabilities for DwGF and DWFG had effects on ΔE Other parameters have limited impacts on ΔC or ΔE

### Probabilistic sensitivity analysis

**Fig 4:**
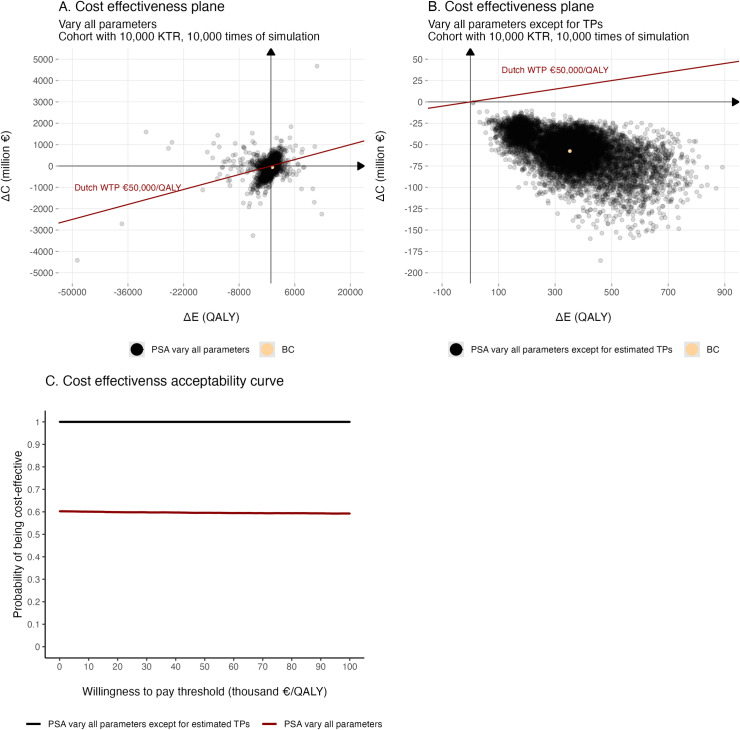
Probabilistic sensitivity analysis. The cost-effectiveness planes show the PSA results of (A) varying all parameters and (B) varying all parameters except for the estimated parameters which calculated transition probabilities. (C) The resultant probabilities of the intervention being cost-effective at differing willingness to pay thresholds are plotted. KTR, kidney transplant recipients; ΔC incremental costs; ΔE incremental effectiveness; PSA, probabilistic sensitivity analysis; TP, transition probability, specifically with the endpoints of graft failure, death with functioning graft and death with graft failure; BC, base case; WTP, willingness to pay; QALY, quality adjusted life year.

The cost-effectiveness plane displays scattered iteration outcomes around the origin (i.e., ΔC=0,ΔE=0 Fig 4A). The probability of being cost-effective is 84.27%. When applying a WTP threshold of €50,000/QALY, the CEAC shows that 59.55% of the outcomes are cost-effective. Alternatively, when all parameters vary except for the parameters used to calculate transition probabilities for GF, DWFG and DwGF, the intervention is suggested to be 100% cost-effective (Fig 4B-C, results of varying one TP parameter at a time see S5 Fig).

### Scenario analysis

**Fig 5:**
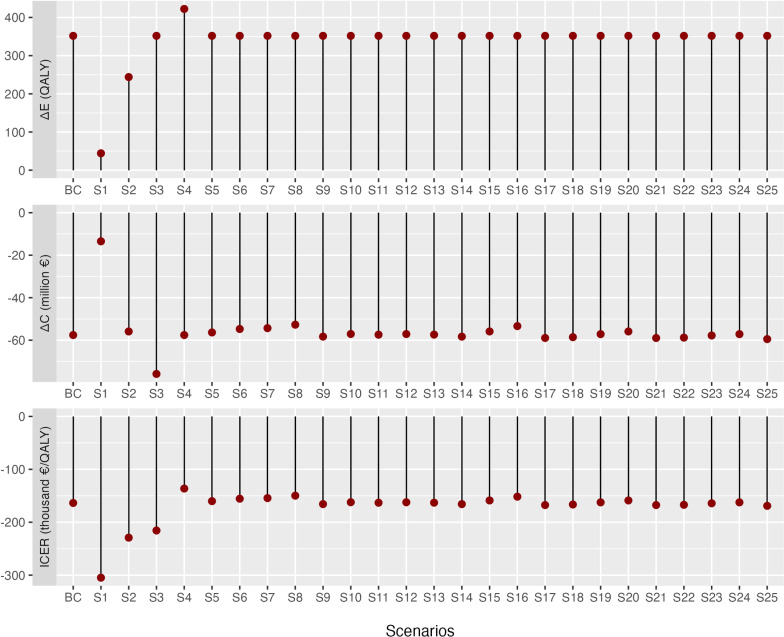
Visualization of the base case and scenario results. ΔE incremental effectiveness; QALY, quality adjusted life year; ΔC incremental costs; BC, base case.

All scenarios suggest the intervention’s dominance (Fig 5). An increased time horizon results in higher incremental QALYs and higher savings (i.e., 10, 20 and 40 years), with an observed small gap of ΔC between 20 and 40 years, indicating that most QALY gains and savings occur in the first 20 years of the model. Overall, the strategy variations in chelation therapy did not influence the dominance.

### Budget impact analysis

Initial costs for the intervention amount to €3.22 million, decreasing drastically to €1.92 million in the second year and further down to about €0.10 million from the third year onward (Table 4). In the first year, the health state-related costs match the ones with the standard of care but rise more gradually in the intervention arm. Generally, the intervention can incur €2.36 million additional expenses in the first year, transitioning to cost savings and increased savings from the second year onwards. In the first five years, €27.12 million can be saved.

**Table 4:** Budget impact over five-year time horizon in the Netherlands (million €).

	Cost category	Year 1	Year 2	Year 3	Year 4	Year 5	Total
Screening and implementing chelation therapy	Health state-related	€165.87	€173.52	€183.81	€194.62	€203.45	€921.27
	Screening and treatment-related	€3.22	€1.92	€0.39	€0.39	€0.39	€6.31
	Total	€169.10	€175.44	€184.20	€195.00	€203.84	€927.58
Standard of care	Health state-related	€166.74	€177.28	€191.23	€204.67	€214.78	€954.70
	Screening and treatment-related	€0.00	€0.00	€0.00	€0.00	€0.00	€0.00
	Total	€166.74	€177.28	€191.23	€204.67	€214.78	€954.70
	**Budget impact**	**€2.36**	**−€1.83**	**−€7.03**	**−€9.67**	**−€10.95**	**−€27.12**

### Value of information

**Fig 6:**
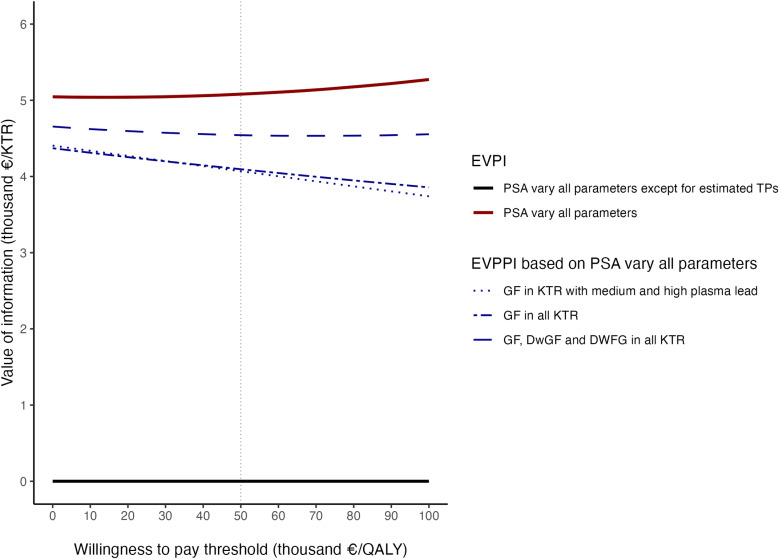
EVPI and EVPPI by willingness to pay. EVPI, expected value of perfect information; EVPPI, expected value of partial perfect information; PSA, probabilistic sensitivity analysis; TP, transition probability; GF, graft failure; DwGF, death with graft failure; DWFG, death with functioning graft; KTR, kidney transplant recipient; QALY, quality adjusted life year.

Under the WTP of €50,000/QALY, the expected value of obtaining perfect information is €5,081/KTR (Fig 6, EVPI). To eliminate the uncertainties around the transition probabilities for all endpoints (i.e., GF, DwGF and DWFG), €4,542/KTR are required, when €4,095/KTR and €4,069/KTR are respectively needed to reduce the uncertainties in transition probabilities for GF among all KTR and KTR in medium and high groups (Fig 6, EVPPI). Across a sample size of zero to 1,000, gathering new information on the meanlogs—the parameter used to calculate transition probabilities for GF in medium and high groups—is estimated to provide an added benefit of €2,261–€2,521/KTR and €1,745–€1,950/KTR correspondingly (S6 Fig).

## Discussion

The study explored the economic feasibility and considerations of screening for and implementing chelation therapy with oral DMSA in KTR with high-normal plasma lead concentrations (referred to as “the intervention” below) to prolong graft functioning in the Netherlands. Our results suggest potential cost savings and health gains from the intervention, which is estimated to save around €27.12 million in the first five years of its launch compared to standard of care. A cap of about €5,000/KTR could eliminate all uncertainties, encouraging further research on the efficacy and administration strategy of oral DMSA.

In the base case, the intervention is dominant compared to the standard of care. However, the DSA reveals uncertainties around the transition probabilities, substantially influencing the cost-effectiveness results. This uncertainty could be partially attributed to the limited number of KTR observations in the parametric survival analysis for the GF endpoint resulting from trichotomization. Additionally, the use of real-world data could cause variations across all endpoints due to clinical complexities. Nevertheless, other parameters, such as costs of center haemodialysis, discounting rate of costs, and utility at the state of functioning graft, while contributing some variations, have moderate impacts on the cost-effectiveness. The extreme variations in the price of DMSA capsules (50% reduction to 200% increase) introduce minimal uncertainties.

PSA indicates that approximately 60% of iteration outcomes are cost-effective. To investigate the reason for the relatively low probability, we re-ran PSA by fixing the base-case values to the transition probabilities for all endpoints. The generated results, in line with DSA, show that whilevariations in other factors, such as direct and indirect costs, systematic considerations, and utility barely influence the intervention’s dominance, variations in transition probabilities are primary contributors to high ICER uncertainty. The transition probabilities for GF in the medium group and DwGF can expand the ICER cloud from the desirable (ΔE>0,ΔC<0) to the undesirable quadrants (ΔE<0), when the transition probability for DWFG mainly affects ΔE and potentially drives the ICER cloud towards the first quadrant (ΔE>0,ΔC>0), thereby affecting the probability of the intervention being cost-effective. Additionally, scenario analyses suggest that the varying strategies of chelation therapy have limited impacts on the cost-effectiveness. Although the time horizon can modify ΔE and ΔC, the intervention remains dominant possibly due to declining marginal costs and effectiveness—as follow-up time extends, ageing KTR may experience reduced utilities, which were not accounted for in the current model.

Our early economic evaluation presents a plausible probability of the intervention being cost-effective. BIA suggests €27.12 million in savings in the first five years from the intervention compared to standard of care. The initial investment is estimated at around €3 million, primarily due to the first scaled screening, which is moderate compared to the health state-related costs. In the following years, the intervention costs plummet.

Synthesizing all available data and our results, the extreme price of DMSA capsules, seemingly flat CEAC curve and additional PSA inspections confirm the neglectable costs surrounding the intervention, offset by the potential savings from delayed dialysis and avoided transplantation. However, uncertain survival rates for all endpoints, particularly GF, are the main concerns, as they could substantially diminish the probability of the intervention’s cost-effectiveness, which currently still hovers around 60%. The desirability of further research to eliminate the aforesaid uncertainties depends on whether study costs remain below the thresholds revealed by VOI: approximately €4,000/KTR to gain perfect information on GF survival (€6,000/KTR if only in medium and high groups, i.e., = 4,000/2  ×  3), €4,500/KTR on GF, DWFG and DwGF (€6,750/KTR), and €5,000/KTR (€7,500/KTR) for all uncertainties. Besides, added benefits vary mildly with sample sizes, suggesting a trial, at a small scale, with a preferably long follow-up period, and examining survivals at all endpoints—specifically, the efficacy of oral DMSA in preserving graft function through lowered lead—could already address key uncertainties exhibited in this study and improve decision-making.

### Future perspectives

Grounded in the study explorations and results, future trials might consider or test the following aspects:

1. *Target population: timing of the intervention*. Our study utilized a KTR cohort. Since the damage caused by elevated lead in kidneys is not specific to KTR, the target population of the intervention could be extended to end-stage CKD patients who share similar experiences as KTR or even earlier stages of CKD where delaying progression to graft failure is vital.
2. *Efficacy and optimal administration of chelation therapy using oral DMSA*. Multiple factors determine the efficacy of oral DMSA. In addition, structural investigations into administration strategies can secure a delicate balance between maximizing graft retention and minimizing potential harms and costs.
  2.1 *Lead threshold for using and stopping the intervention*. While Sotomayor et al. (2022) have hinted a plasma lead concentration above 0.32 µg/L increases the risks of graft failure [11], a conservative threshold of 0.38 µg/L from the publication’s trichotomization was adopted. Nevertheless, there is no universally agreed threshold.
  2.2 *Minimally effective dosage and duration of chelating effects*. “Effective” dosage incorporates 1) efficacy in chelating lead under the desirable threshold given normal but elevated lead and 2) efficacy in slowing the progression to graft failure, which are the survival rates associated with GF, DwGF and DWFG in intervention and non-intervention arms. “Duration” of chelating effects encompasses 1) the time required for lead redistribution and lowered to the desirable threshold, and 2) the time after repeated chelation therapy until re-exposure to lead above the desirable threshold. These factors are crucial in adopting oral DMSA in our intended setting, designing administration strategies and screening regimes and establishing protocols for repeated chelation therapy.
  2.3 *Possible additional effects due to cadmium removal*. Chronic lead and cadmium exposures often co-occur [11,56,57]. Compared to lead, cadmium is more detrimental in damaging kidneys [10,11]. Animal studies have provided evidence that DMSA can chelate cadmium [17], but scarce evidence in humans [17,58], absent clinical application, and unknown lead-cadmium interaction have limited our study to lead only. Potential added value from cadmium removal thus should not be ruled out. Furthermore, *in vitro* and *in vivo* studies have discovered another promising chelator for cadmium—monoisoamyl meso-2,3-dimercaptosuccinic acid (MiADMSA), a monoester analogue of DMSA [59], and suggested a combinatory use of DMSA and MiADMSA to increase chelating efficacy [59–61], although yet to be confirmed by human studies. Therefore, explorations on the chelating effect of oral DMSA and its complementary use with MiADMSA on cadmium could be beneficial. Subsequent complications exemplified by the cadmium threshold and its interaction with lead require handling to maximize the intervention’s utility by design.
3. *Dynamic of lead under chronic exposure and intervention*. Chronic lead exposure tends to accumulate with age and inadvertently poses long-term health risks. While this study assumed lead stability based on the study setting and short-term evidence, understanding the natural trajectory of lead accumulation and the potential for interventions to disrupt, redistribute, and rebalance lead levels over time is essential to inform a precise design of intervention strategies.
4. *Initial and follow-up screenings: interval and timeframe*. Target population, long-term effects of oral DMSA and lead dynamics can indicate the interval and timeframe of screening. The optimal design, meanwhile, should consider marginal benefits versus costs, for instance, incorporating potential post-transplantation complications with earlier screening and ageing along an extended timeframe.
5. *Other considerations*.
  5.1 *Data collection*. Despite our strict compliance with the Dutch guidelines, the used utilities reported by Liem et al. (2008) and generated from EQ-5D-3L might be outdated, though the best available evidence. Collecting EQ-5D-5L is needed due to the technological development that has enabled different experiences around dialysis and transplantation and Dutch guidelines’ suggestion on using EQ-5D-5L. Besides, the recent lack of data and insights into disparate patient journeys under two treatment schemes (i.e., standard of care and the intervention) has hindered a comprehensive incorporation of associated “societal” costs. Therefore, patient journeys should be well-understood, and their relevant costs, such as informal care, should be identified and collected for future evaluation.
  5.2 *Institutional context*. To guide future research in the Dutch context, our study is tailored to the Netherlands, whose results may apply to neighboring countries such as Germany. However, the intervention might offer increased benefits in developing countries, considering key factors including 1) more heavy metal exposures due to limited regulations, 2) lower cost of chelation therapy compared to kidney replacement therapy, 3) a larger at-risk population (e.g., high CKD prevalence and substantial population size), and 4) resource constraints (e.g., limited healthcare access, long transplant waiting times, and restricted technology availability).

Given the intricacies and uncertainties of the intervention, dependent on available infrastructures and resources, future trials might benefit from additional preclinical studies. Leveraging existing cohorts and registry data across regions, supplemented by measurement of plasma lead concentration, in-depth and large-scale investigations into the lead threshold and corresponding survival rates would be possible, thus refining the range for further trial exploration. In short, gathering empirical evidence, especially through (long-term) clinical studies, is indispensable for validating the model’s hypotheses and assumptions and updating follow-up evaluations on the intervention. Only with such dedicated endeavors can the likelihood of shaping policies, updating guidelines, and realizing the intervention in clinical practices be enhanced.

### Strengths and limitations

To our knowledge, we are the first to systematically explore the economic feasibility of screening for and implementing chelation therapy using oral DMSA in KTR with high-normal plasma lead concentrations. We utilized real-world data from a large cohort spanning 2011 to 2020 and representative of the Dutch KTR population. We applied NICE guidance to calculate and extrapolate survival probabilities in different health states [36]. We explored various scenarios to address the uncertainties surrounding the administration strategies. Our early economic evaluation, a meticulous and rigorous assessment drawing upon the existing body of evidence, highlights the potential value of integrating chelation therapy into post-kidney transplantation routines and emphasizes the need for research to substantiate the true benefits of DMSA capsules among individuals with an elevated but normal level of lead in maintaining graft function. Importantly, we outlined key considerations to guide future endeavors.

Despite its strengths, the study has several limitations. Firstly, utilities calculated from EQ-5D-3L were used, deviating slightly from the Dutch guidelines, and were not age-adjusted due to insufficient evidence. Nonetheless, delaying progression to graft failure remains preferable from a patient’s perspective. Secondly, including blood tests as separate costs may overestimate expenses, as chelation therapy could coincide with routine post-transplantation check-ups [62]. This decision, however, accounts for possible variations in the time of routine checks and flexibility in each step of chelation therapy. Thirdly, Markov models cannot track individual patients, meaning that KTR in the high group who surpass the treatment threshold at certain time points are unidentifiable, and transition probabilities thus could not be adjusted. This could cause a slight overestimation of effectiveness and explain the constant incremental effectiveness in scenario analyses. Fourthly, alternative DMSA access was unexplored (i.e., pharmacies purchasing raw materials from manufacturers to compound DMSA capsules) owing to data unavailability, though our results were insensitive to DMSA prices. Furthermore, our conservative approach—targeting KTR, applying a 0.38 µg/L threshold, lowering lead from high to medium group and excluding cadmium removal—has yielded modest QALY gains (0.106 QALY gains per KTR eligible for chelation therapy, i.e., = 351.88/10,000  × 3). This approach potentially underestimates overall effectiveness but ensures the study validity and is thereby preferred given the study aims. Lastly, due to the limited research on the intended intervention in this new setting, our assumptions were informed by expert opinions, current guidelines and publications. Although the number of assumptions implies that our study is primarily rooted in theoretical exploration, our study offers a robust foundation for subsequent empirical investigations, aligned with the objectives of early HTA to guide future research and support the development of the intervention.

## Conclusions

Preserving kidney function is paramount. In this context, our early economic evaluation suggests that screening for and implementing chelation therapy with oral DMSA in KTR exhibiting high-normal plasma lead concentrations is likely cost-effective compared to the standard of care, saving costs and improving health. The cost-effectiveness is primarily driven by (graft) survival probabilities while remaining robust against other uncertainties. The proposal of applying chelation therapy to individuals with normal-high lead to prevent graft failure is at an early stage but demonstrates substantial potential. Therefore, there is an imperative need for trials that systematically evaluate the efficacy and administration protocol of chelation therapy, including the optimal treatment threshold, addressing current gaps in evidence.

## Supporting information

S1 Table CHEERS 2022 checklist. (DOCX)

S2 Table Baseline characteristics of the UMCG cohort. (DOCX)

S3 Table Assumptions, rationales, and impacts on results. (DOCX)

S4 Table Estimated model parameters. (DOCX)

S1 Fig Distribution of plasma lead concentration. (DOCX)

S2 Fig Transition probabilities of different endpoints. (DOCX)

S3 Fig Deterministic sensitivity analysis on incremental costs with all parameters listed. (DOCX)

S4 Fig Deterministic sensitivity analysis on incremental effectiveness with all parameters listed. (DOCX)

S5 Fig Probabilistic sensitivity analysis focusing on transition probabilities. (DOCX)

S6 Fig Expected value of sample information. (DOCX)
