# Supplementary material for: Early economic evaluation of chelation therapy in kidney transplant recipients with high-normal lead
Source: PLoS One. 2025 Feb 27;20(2):e0319022. doi: 10.1371/journal.pone.0319022 (PMC11867398; doi:10.1371/journal.pone.0319022)
Supplement: S1 Table — (DOCX) [file pone.0319022.s001.docx]

## S1 Table. CHEERS 2022 checklist.

| Topic | No. | Item | Location where item is reported |
| --- | --- | --- | --- |
| Title |  |  |  |
|  | 1 | Identify the study as an economic evaluation and specify the interventions being compared. | Title |
| Abstract |  |  |  |
|  | 2 | Provide a structured summary that highlights context, key methods, results, and alternative analyses. | Abstract |
| Introduction |  |  |  |
| Background and objectives | 3 | Give the context for the study, the study question, and its practical relevance for decision making in policy or practice. | Introduction, Paragraph 4 |
| Methods |  |  |  |
| Health economic analysis plan | 4 | Indicate whether a health economic analysis plan was developed and where available. | Material and Methods, Paragraph 1 |
| Study population | 5 | Describe characteristics of the study population (such as age range, demographics, socioeconomic, or clinical characteristics). | Material and Methods, Patient cohort and S2 Table |
| Setting and location | 6 | Provide relevant contextual information that may influence findings. | Material and Methods, Patient cohort and Discussion, Future perspective |
| Comparators | 7 | Describe the interventions or strategies being compared and why chosen. | Material and Methods, Standard of care and chelation therapy, Paragraph 1 |
| Perspective | 8 | State the perspective(s) adopted by the study and why chosen. | Material and Methods, Costs, Paragraph 1, Line 1 |
| Time horizon | 9 | State the time horizon for the study and why appropriate. | Material and Methods, Model description, Line 1-2 |
| Discount rate | 10 | Report the discount rate(s) and reason chosen. | Material and Methods, Table 1 |
| Selection of outcomes | 11 | Describe what outcomes were used as the measure(s) of benefit(s) and harm(s). | Material and Methods, Utilities |
| Measurement of outcomes | 12 | Describe how outcomes used to capture benefit(s) and harm(s) were measured. | Material and Methods, Transition probabilities and Utilities |
| Valuation of outcomes | 13 | Describe the population and methods used to measure and value outcomes. | Material and Methods, Transition probabilities and Utilities |
| Measurement and valuation of resources and costs | 14 | Describe how costs were valued. | Material and Methods, Costs |
| Currency, price date, and conversion | 15 | Report the dates of the estimated resource quantities and unit costs, plus the currency and year of conversion. | Material and Methods, Costs, Paragraph 1, Line 2-3 |
| Rationale and description of model | 16 | If modelling is used, describe in detail and why used. Report if the model is publicly available and where it can be accessed. | Material and Methods, Model description |
| Analytics and assumptions | 17 | Describe any methods for analysing or statistically transforming data, any extrapolation methods, and approaches for validating any model used. | Relevant assumptions can be found in each corresponding Material and Methods section |
| Characterising heterogeneity | 18 | Describe any methods used for estimating how the results of the study vary for subgroups. | Not applicable |
| Characterising distributional effects | 19 | Describe how impacts are distributed across different individuals or adjustments made to reflect priority populations. | Not applicable |
| Characterising uncertainty | 20 | Describe methods to characterise any sources of uncertainty in the analysis. | Material and Methods, Sensitivity analyses |
| Approach to engagement with patients and others affected by the study | 21 | Describe any approaches to engage patients or service recipients, the general public, communities, or stakeholders (such as clinicians or payers) in the design of the study. | Not applicable |
| Results |  |  |  |
| Study parameters | 22 | Report all analytic inputs (such as values, ranges, references) including uncertainty or distributional assumptions. | Material and Methods, Table 1 |
| Summary of main results | 23 | Report the mean values for the main categories of costs and outcomes of interest and summarise them in the most appropriate overall measure. | Results, Cost-effectiveness analysis |
| Effect of uncertainty | 24 | Describe how uncertainty about analytic judgments, inputs, or projections affect findings. Report the effect of choice of discount rate and time horizon, if applicable. | Results, Deterministic sensitivity analysis; Probabilistic sensitivity analysis; and Scenario analysis |
| Effect of engagement with patients and others affected by the study | 25 | Report on any difference patient/service recipient, general public, community, or stakeholder involvement made to the approach or findings of the study | Results, Value of information |
| Discussion |  |  |  |
| Study findings, limitations, generalisability, and current knowledge | 26 | Report key findings, limitations, ethical or equity considerations not captured, and how these could affect patients, policy, or practice. | Discussion |
| Other relevant information |  |  |  |
| Source of funding | 27 | Describe how the study was funded and any role of the funder in the identification, design, conduct, and reporting of the analysis | End of manuscript |
| Conflicts of interest | 28 | Report authors conflicts of interest according to journal or International Committee of Medical Journal Editors requirements. | End of manuscript |

The checklist is based on the publication[1].

## Reference

1. Husereau D, Drummond M, Augustovski F, de Bekker-Grob E, Briggs AH, Carswell C, et al. Consolidated Health Economic Evaluation Reporting Standards (CHEERS) 2022 Explanation and Elaboration: A Report of the ISPOR CHEERS II Good Practices Task Force. Value Health. 2022;25(1):10-31.
