# Supplementary material for: Early economic evaluation of chelation therapy in kidney transplant recipients with high-normal lead
Source: PLoS One. 2025 Feb 27;20(2):e0319022. doi: 10.1371/journal.pone.0319022 (PMC11867398; doi:10.1371/journal.pone.0319022)
Supplement: S2 Table — (DOCX) [file pone.0319022.s002.docx]

## S2 Table. Baseline characteristics of the UMCG cohort.

|  | All |  | Tertiles by plasma lead concentrations | | | | |
| --- | --- | --- | --- | --- | --- | --- | --- |
|  |  |  | **Low** |  | **Medium** |  | **High** |
|  |  |  | (≤ 0.24 µg/L) |  | (0.24-0.38 µg/L) |  | (≥ 0.38 µg/L) |
| Characteristics (mean/SD; count/%) | N = 670 |  | N = 221 |  | N = 224 |  | N = 225 |
| Sex, female | 285 (42.5%) |  | 117 (52.9%) |  | 79 (35.3%) |  | 89 (39.6%) |
| Age, year | 53.02 (12.75) |  | 49.57 (12.84) |  | 53.31 (12.53) |  | 56.13 (12.09) |
| Weight, kg | 80.69 (16.45) |  | 78.83 (16.62) |  | 82.33 (15.89) |  | 80.87 (16.72) |
| BMI, kg/m^2^ | 26.71 (4.78) |  | 26.31 (5.13) |  | 26.88 (4.24) |  | 26.92 (4.92) |
| Diabetes status, yes | 161 (24.0%) |  | 57 (25.8%) |  | 52 (23.2%) |  | 52 (23.1%) |
| Smoking status |  |  |  |  |  |  |  |
| Never | 266 (39.7%) |  | 107 (48.4%) |  | 87 (38.8%) |  | 72 (32.0%) |
| Previously | 284 (42.4%) |  | 80 (36.2%) |  | 95 (42.4%) |  | 109 (48.4%) |
| Yes | 80 (11.9%) |  | 23 (10.4%) |  | 29 (12.9%) |  | 28 (12.4%) |
| Unknown | 40 (6.0%) |  | 11 (5.0%) |  | 13 (5.8%) |  | 16 (7.1%) |
| Alcohol consumption |  |  |  |  |  |  |  |
| 0 g/d | 60 (9.0%) |  | 15 (6.8%) |  | 20 (8.9%) |  | 25 (11.1%) |
| 0-10 g/d | 383 (57.2%) |  | 141 (63.8%) |  | 121 (54.0%) |  | 121 (53.8%) |
| 10-30 g/d | 130 (19.4%) |  | 36 (16.3%) |  | 54 (24.1%) |  | 40 (17.8%) |
| > 30 | 30 (4.5%) |  | 5 (2.3%) |  | 12 (5.4%) |  | 13 (5.8%) |
| Unknown | 67 (10.0%) |  | 24 (10.9%) |  | 17 (7.6%) |  | 26 (11.6%) |
| Donor type, living | 231 (34.5%) |  | 93 (42.1%) |  | 80 (35.7%) |  | 58 (25.8%) |
| Number of kidney transplants |  |  |  |  |  |  |  |
| 1 | 603 (90.1%) |  | 201 (91.0%) |  | 203 (91.0%) |  | 199 (88.4%) |
| 2 | 61 (9.1%) |  | 17 (7.7%) |  | 19 (8.5%) |  | 25 (11.1%) |
| 3 | 4 (0.6%) |  | 2 (0.9%) |  | 1 (0.4%) |  | 1 (0.4%) |
| 4 | 1 (0.1%) |  | 1 (0.5%) |  | 0 (0.0%) |  | 0 (0.0%) |
| Pre-emptive kidney transplants, yes | 106 (15.8%) |  | 55 (24.9%) |  | 27 (12.1%) |  | 24 (10.7%) |
| Transplant vintage, year | 8.04 (7.63) |  | 9.24 (8.49) |  | 8.29 (7.04) |  | 6.63 (7.10) |
| eGFR, ml/min/1.73 m^2^ | 52.48 (20.10) |  | 54.78 (20.77) |  | 50.32 (20.05) |  | 52.37 (19.31) |
| Urinary protein excretion, g/24 hr | 0.40 (0.85) |  | 0.35 (0.80) |  | 0.43 (0.80) |  | 0.43 (0.95) |
| Plasma cadmium, µg/L | 0.07 (0.13) |  | 0.06 (0.05) |  | 0.07 (0.03) |  | 0.09 (0.22) |
| Plasma lead, µg/L | 0.39 (0.30) |  | 0.19 (0.03) |  | 0.31 (0.04) |  | 0.66 (0.39) |

Abbreviations: SD, standard deviation; BMI, body mass index; eGFR, estimated glomerular filtration rate; UMCG, University Medical Center Groningen.
