# Supplementary material for: Early economic evaluation of chelation therapy in kidney transplant recipients with high-normal lead
Source: PLoS One. 2025 Feb 27;20(2):e0319022. doi: 10.1371/journal.pone.0319022 (PMC11867398; doi:10.1371/journal.pone.0319022)
Supplement: S3 Table — (DOCX) [file pone.0319022.s003.docx]

## Table S3 Key assumptions, rationales and impacts

| **Assumptions in BC** | | **Rationales** | **Impacts on results** |
| --- | --- | --- | --- |
| **General** | | | |
| a6 | Lead stability: all patients remain in the same group except for the patients eligible for oral DMSA transitoning from high (medium) to medium (high) due to chelation therapy (re-exposure) | There are three reasons: 1) in the general population, cumulative lead toxicant could increase with age. However, this age effect might be limited because nowadays, developed countries, including the Netherlands (i.e., our study setting), usually have stricter regulations and thus relatively more controlled exposures. 2) Using the same Dutch KTR cohort, Sotomayor et al. have found that the plasma lead concentration is stable minimally for two years[1]. Lastly, 3) the mean age of patients was 53 in the used Dutch KTR cohort, which is representative of the Dutch nationwide KTR population, and the hypothetical cohort used for modelling. If a patient's lifestyle, occupational exposures and living environment have not resulted in a slightly elevated plasma lead concentration for the first and over 50 years of life, logically, the possibility of this patient transitioning to a high-normal level in lifetime and consequently requiring chelation therapy is mostly neglectable. | Mostly neglectable. However, we acknowledge that the relative stability of lead is an assumption that might carry slight uncertainties and requires future studies. |
| **Intervention stage: first screening for eligible patients** | | | |
|  | Time: first regular checkup post-transplantation | Convenient as an add-on to the current standard of care (i.e., regular outpatient checkups at a three-month interval). | Neglectable. |
|  | Target population: all incidental KTR | The cohort used for this study only includes KTR. | Conservative results were derived, although justifiable and preferred given the context. Target population could be potentially extended to CKD patients. |
| **Intervention stage: chelation therapy provided to eligible patients** | | | |
| a1 | Target population: KTR in the high group (intervention threshold 0.38 µg/L) | Therer is no universally agreed cut-off. Based on the findings by Sotomayor et al.[1], the same method of “trichotomization” was used. Considering the fact that this study is an early HTA with limited evidence, the use of oral DMSA should be restricted to essential cases to balance the interventional benefits and safety. Therefore, only KTR with high-normal plasma lead concentration—an intervention threshold 0.38 µg/L—are regarded eligible for oral DMSA. | The choice of using possibly conservative threshold might result in an underestimation of treatment benefits, which however, is justifiable and preferred given the context. Because overestimating the effect by including additional patients who may benefit from the intended intervention could lead to false positive results, risking prospective research. |
| a2 | Efficacy: from high to medium group only | A conservative choice. The same rationales as stated in a1. | Conservative. The same impact direction as stated in a1. |
| a3 | Side effects: no adverse event | From case reports and literature, general side effects of oral DMSA include the following[2]:   1. Impacts on other metals: however, inconclusive; 2. Transaminase activity: however, do not result in clinically significant sequelae, suggesting reversibility; 3. Skin reactions: likely to occur during multiple courses and can be naturally resolved after treatment discontinuation; 4. Other reported adverse effects: 1) nausea (N=9) might be due to the lead redistribution, and 2) hemolytic anemia (N=1) were naturally resolved after treatment discontinuation.   In our study, considering the use of low dosage and vulnerabilities of KTR, the potential adverse effects could be adjusted accordingly, leaving e1) impacts on other metals and e2) transaminase activities as the main concerns. To elaborate further, oral DMSA targets KTR with high-normal plasma lead concentration. In the base case, the yearly intervention consists of one course of chelation therapy, a five-day intake with a low dosage (i.e., 10 mg/kg/day)—one-third of the recommended dosage for lead poisoning within one course. Overall, the combination of low dosage and single course is most likely to be safe. | Scenario analyses by including two safety measures (i.e., checking for iron deficiency and liver dysfunction in response to the first two potential side effects above) suggest minimal differences in costs when including and excluding the safety measures. In combination of the study results, we only expect our assumptions regarding adverse effects to underestimate costs or health risks to a minimal extent. |
| a4 | Period of efficacy: at least five years since the first course of oral DMSA | A previous study by Lin-Tan et al. suggested that “…repeated chelation therapies, over a four-year period, slow progression of renal insufficiency…”, although the trial focused on CaEDTA, an earlier chelating agent to DMSA[3]. Since DMSA has demonstrated improved safety and chelating efficacy, we assumed a five-year duration. | Scenario analyses that tested period of efficacy from five years to lifetime suggest neglectable changes in costs. |
| **Intervention stage: follow-up screenings in case of re-exposure** | | | |
| a5 | Time: every five years | This is an assumption based on a4. Rationales see a4. | Impacts see a4. |
| a5 | Target population: only the patients in high group at the first screening | This is an assumption based on a6. Rationals see a6. | Impacts see a6. |
| a5 | Re-exposure rate: 10% of the eligible patients | On top of the relative lead stability (rationales see a6), considering that lead levels within the normal range are expected to accumulate slowly, we set the re-exposure assumption at 10%. | Scenario analyses that varied the re-exposure rate for repeated intervention among at-risk KTR from 1–60% suggest neglectable changes in costs. |

Abbreviations: BC, base case; KTR, kidney transplant recipient; CKD, chronic kidney diseases; HTA, health technology assessment; DMSA, meso-2,3-dimercaptosuccinic acid; CaEDTA, calcium disodium ethylenediaminetetraacetic acid; NA, not applicable.

## Reference

1. Sotomayor CG, Giubergia F, Groothof D, Ferreccio C, Nolte IM, Navis GJ, et al. Plasma Lead Concentration and Risk of Late Kidney Allograft Failure: Findings From the TransplantLines Biobank and Cohort Studies. American Journal of Kidney Diseases. 2022;80(1):87-97.e1. doi: 10.1053/j.ajkd.2021.10.009.

2. Bradberry S, Vale A. Dimercaptosuccinic acid (succimer; DMSA) in inorganic lead poisoning. Clinical Toxicology. 2009;47(7):617-31. doi: 10.1080/15563650903174828.

3. Lin-Tan D-T, Lin J-L, Yen T-H, Chen K-H, Huang Y-L. Long-term outcome of repeated lead chelation therapy in progressive non-diabetic chronic kidney diseases. Nephrology Dialysis Transplantation. 2007;22(10):2924-31. doi: 10.1093/ndt/gfm342.

4. Macdougall IC, Bircher AJ, Eckardt K-U, Obrador GT, Pollock CA, Stenvinkel P, et al. Iron management in chronic kidney disease: conclusions from a “Kidney Disease: Improving Global Outcomes”(KDIGO) Controversies Conference. Kidney international. 2016;89(1):28-39.
