# Supplementary material for: Early economic evaluation of chelation therapy in kidney transplant recipients with high-normal lead
Source: PLoS One. 2025 Feb 27;20(2):e0319022. doi: 10.1371/journal.pone.0319022 (PMC11867398; doi:10.1371/journal.pone.0319022)
Supplement: S4 Table — (DOCX) [file pone.0319022.s004.docx]

## Table S4 Estimated model parameters

| **Endpoint** | **Group** | **Value** | | **Range** | | | **Source** |
| --- | --- | --- | --- | --- | --- | --- | --- |
|  |  |  |  | SE | LCI | UCI |  |
| **GF** |  | **Log-normal parameters** | |  |  |  | UMCG dataset[1] |
|  | High | meanlog = | -0.557 | 0.787 | -2.099 | 0.985 |  |
|  |  | sdlog = | 1.283 | 0.187 | 0.964 | 1.708 |  |
|  | Medium | meanlog = | 1.147 | 0.849 | -0.517 | 2.812 |  |
|  |  | sdlog = | 1.107 | 0.150 | 0.849 | 1.443 |  |
|  | Low | meanlog = | -1.254 | 0.868 | -2.955 | 0.448 |  |
|  |  | sdlog = | 1.267 | 0.170 | 0.973 | 1.649 |  |
| **DWFG** |  | **Gamma parameters** | |  |  |  | UMCG dataset[1] |
|  | All | shape = | 1.610 | 0.160 | 1.325 | 1.956 |  |
|  |  | rate = | 0.139 | 0.022 | 0.102 | 0.189 |  |
| **DwGF** |  | **Gamma parameters** | |  |  |  | UMCG dataset[1] |
|  | All | shape = | 2.113 | 0.476 | 1.358 | 3.287 |  |
|  |  | rate = | 0.236 | 0.074 | 0.128 | 0.436 |  |

Abbreviations: GF, graft failure; DWFG, death with functioning graft; DwGF, death with graft failure; SE, standard error; LCI, lower 95% confidence interval; UCI, upper 95% confidence interval; UMCG, University Medical Center Groningen.

## Reference

1. Eisenga MF, Gomes-Neto AW, Van Londen M, Ziengs AL, Douwes RM, Stam SP, et al. Rationale and design of TransplantLines: a prospective cohort study and biobank of solid organ transplant recipients. BMJ Open. 2018;8(12):e024502.
