# Supplementary material for: Early economic evaluation of chelation therapy in kidney transplant recipients with high-normal lead
Source: PLoS One. 2025 Feb 27;20(2):e0319022. doi: 10.1371/journal.pone.0319022 (PMC11867398; doi:10.1371/journal.pone.0319022)
Supplement: S1 Fig — (DOCX) [file pone.0319022.s005.docx]

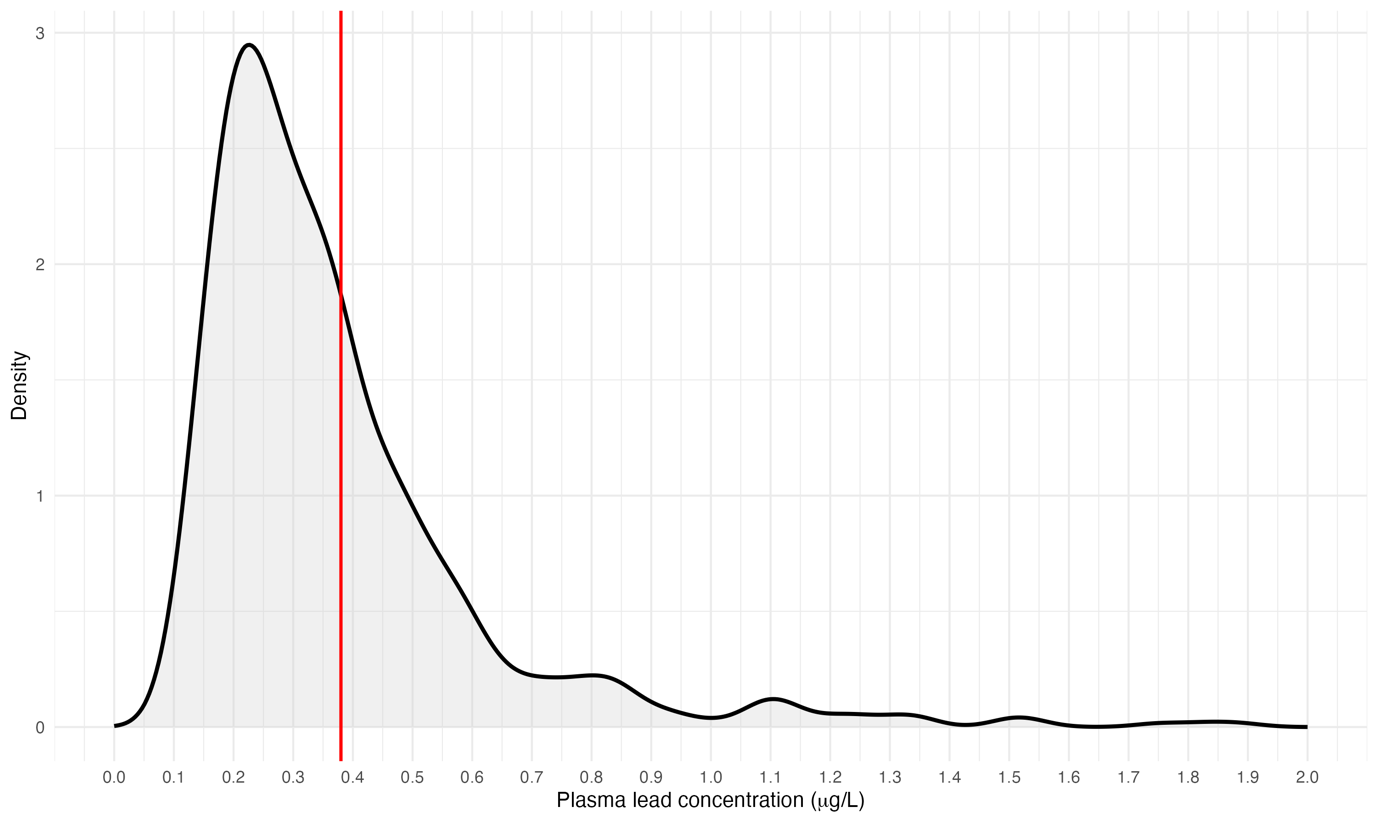


## S1 Fig. Distribution of the plasma lead concentration in the cohort.

The red line represents the eligibility cut-off (= 0.38 µg/L) of the plasma lead concentration for receiving chelation therapy.
