# Supplementary figures and images for: Early economic evaluation of chelation therapy in kidney transplant recipients with high-normal lead
Source: PLoS One. 2025 Feb 27;20(2):e0319022. doi: 10.1371/journal.pone.0319022 (PMC11867398; doi:10.1371/journal.pone.0319022)

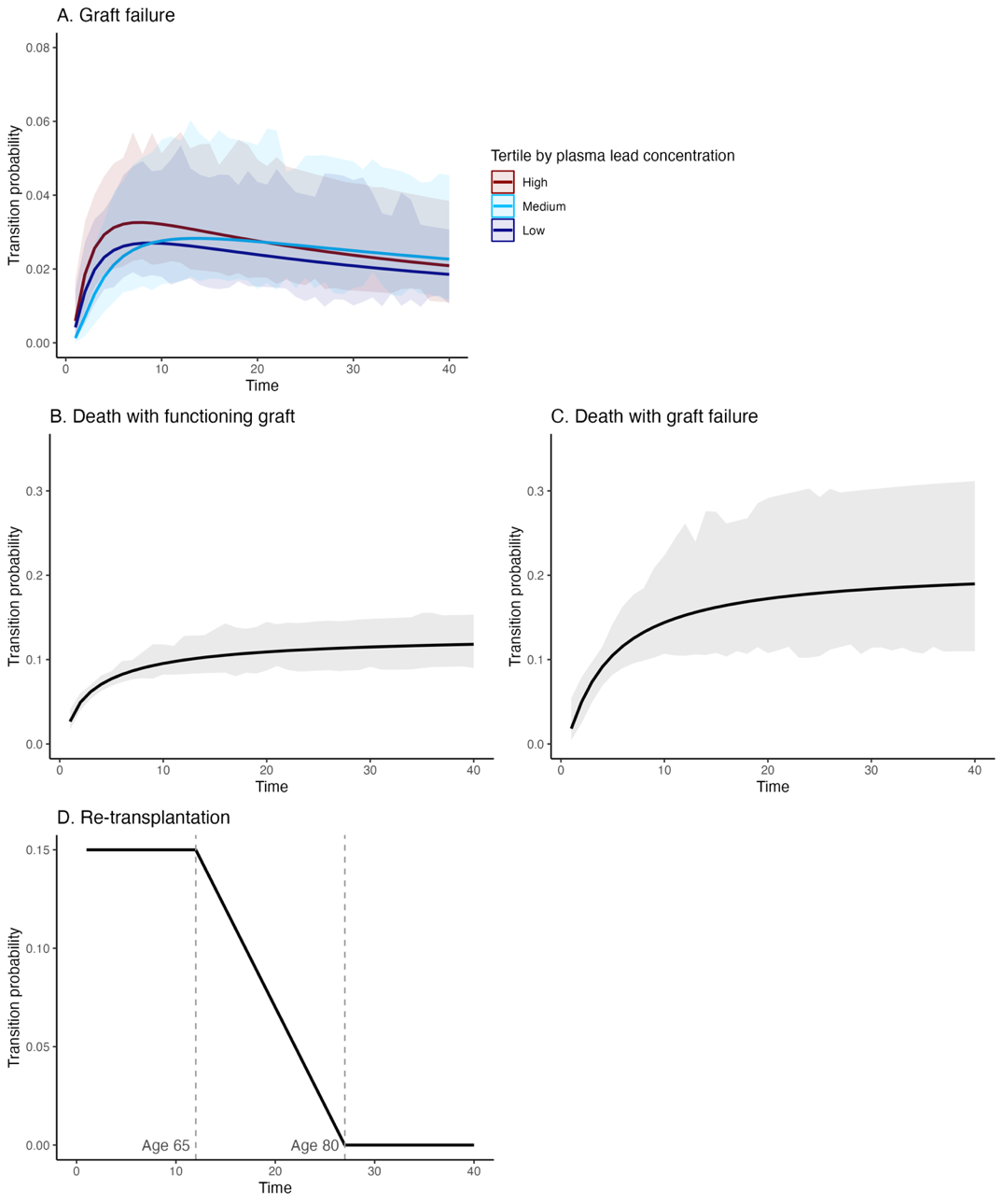


## S2 Fig. Transition probabilities of different endpoints.

Supplement: S2 Fig — (DOCX) [file pone.0319022.s006.docx]
