# Supplementary material for: Early economic evaluation of chelation therapy in kidney transplant recipients with high-normal lead
Source: PLoS One. 2025 Feb 27;20(2):e0319022. doi: 10.1371/journal.pone.0319022 (PMC11867398; doi:10.1371/journal.pone.0319022)
Supplement: S3 Fig — (DOCX) [file pone.0319022.s007.docx]

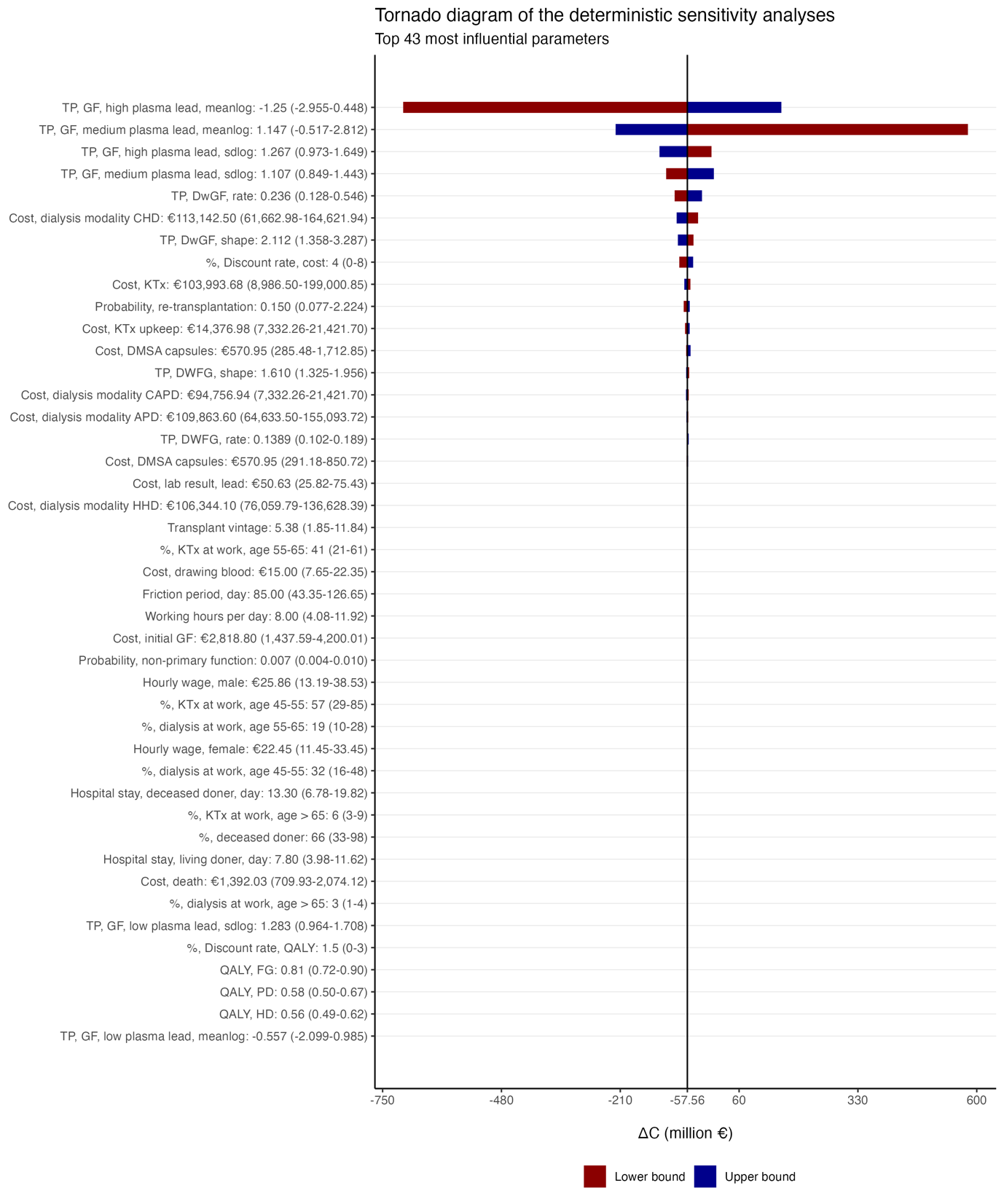


## S3 Fig. Deterministic sensitivity analysis on incremental costs with all parameters listed.

Meanlog and sdlog are the parameters from lognormal distribution and were estimated through the parametric survival analyses. These parameters are used to calculate the transition probabilities of graft failure in different subgroups of KTR with high, medium and low plasma lead concentrations. Rate and shape are the parameters from gamma distribution and were estimated through the parametric survival analyses. These parameters are used to calculate the transition probabilities of death with functioning graft and death with graft failure. Abbreviations: $\Delta C$, incremental costs; TP, transition probability; GF, graft failure; FG, functioning graft; DwGF, death with graft failure; DWFG, death with functioning graft; KTx, kidney transplantation; DMSA, meso-2,3-dimercaptosuccinic acid; HD, haemodialysis; PD, peritoneal dialysis; CHD, center haemodialysis; HHD, home haemodialysis; CAPD, continuous ambulatory peritoneal dialysis; APD, automated peritoneal dialysis; QALY, quality adjusted life year.
