# Supplementary material for: Early economic evaluation of chelation therapy in kidney transplant recipients with high-normal lead
Source: PLoS One. 2025 Feb 27;20(2):e0319022. doi: 10.1371/journal.pone.0319022 (PMC11867398; doi:10.1371/journal.pone.0319022)
Supplement: S5 Fig — (DOCX) [file pone.0319022.s009.docx]

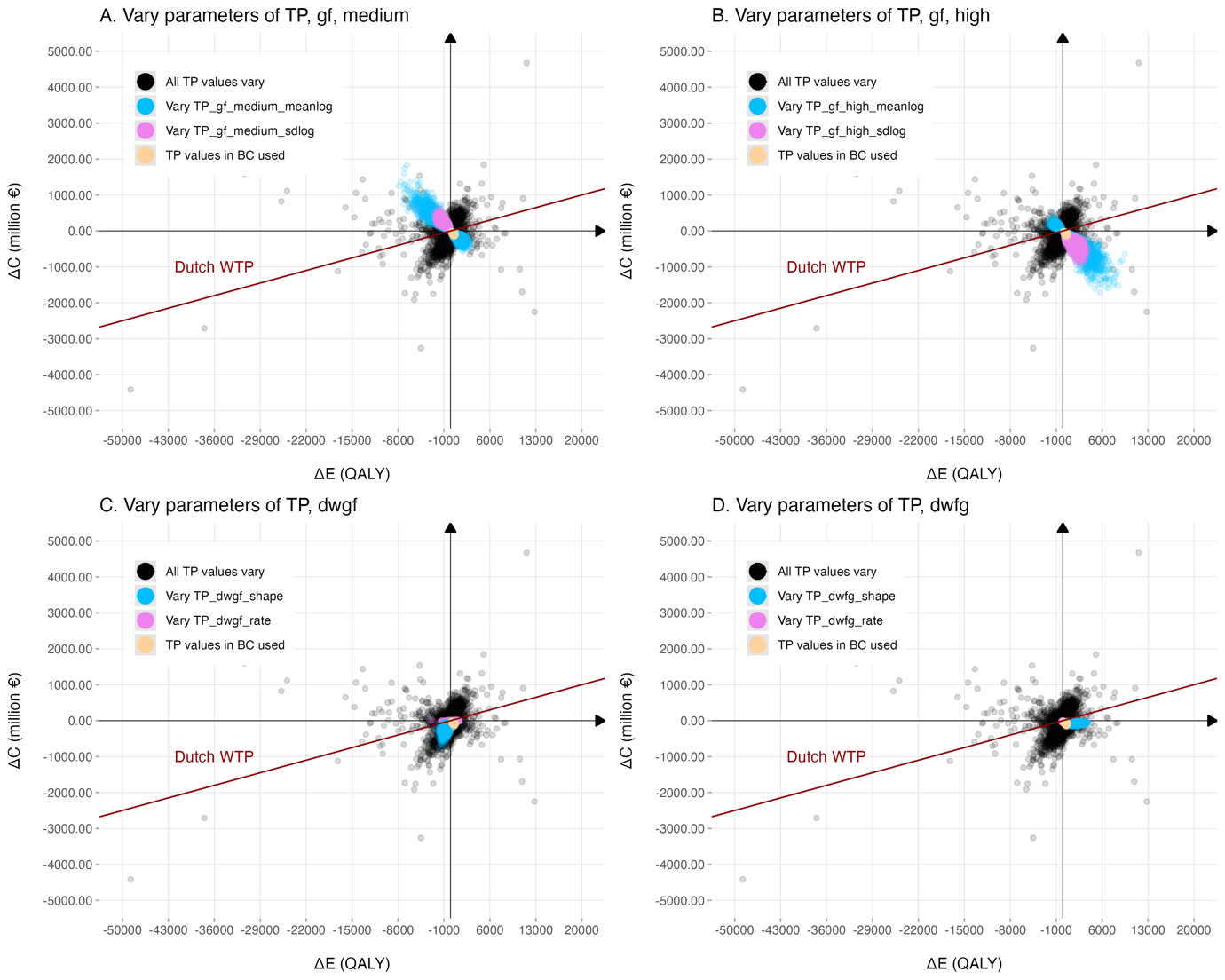


## S5 Fig. Probabilistic sensitivity analysis focusing on transition probabilities.

Meanlog and sdlog are the parameters from lognormal distribution and were estimated through the parametric survival analyses. These parameters are used to calculate the transition probabilities of graft failure in different subgroups of KTR with high, medium and low plasma lead concentrations. Rate and shape are the parameters from gamma distribution and were estimated through the parametric survival analyses. These parameters are used to calculate the transition probabilities of death with functioning graft and death with graft failure. Abbreviations: TP, transition probabilities; GF, graft failure; DWFG, death with functioning graft; DwGF, death with graft failure; BC, base case; WTP, willingness to pay (i.e., €50,000/QALY).
